# *Broa*, an Ethnic Maize Bread, as a Source of Phenolic Compounds

**DOI:** 10.3390/antiox10050672

**Published:** 2021-04-26

**Authors:** Andreia Bento-Silva, Noélia Duarte, Elsa Mecha, Maria Belo, Ana Teresa Serra, Maria Carlota Vaz Patto, Maria Rosário Bronze

**Affiliations:** 1 FCT NOVA, Faculdade de Ciências e Tecnologia, Campus da Caparica, Universidade Nova de Lisboa, 2829-516 Caparica, Portugal; abentosilva@ff.ulisboa.pt; 2 ITQB NOVA, Instituto de Tecnologia Química e Biológica António Xavier, Universidade Nova de Lisboa, Avenida da República, 2780-157 Oeiras, Portugal; emecha@itqb.unl.pt (E.M.); mariabelo87@gmail.com (M.B.); cpatto@itqb.unl.pt (M.C.V.P.); 3 FFULisboa, Faculdade de Farmácia da Universidade de Lisboa, Av. das Forças Armadas, 1649-019 Lisboa, Portugal; 4 iMed.ULisboa, Instituto de Investigação do Medicamento, Faculdade de Farmácia, Universidade de Lisboa, Avenida Prof. Gama Pinto, 1649-003 Lisboa, Portugal; mduarte@ff.ulisboa.pt; 5 iBET, Instituto de Biologia Experimental e Tecnológica, Avenida da República, Quinta-do-Marquês, Estação Agronómica Nacional, Apartado 12, 2780-157 Oeiras, Portugal; tserra@ibet.pt

**Keywords:** maize, *broa*, ferulic acid, *p*-coumaric acid, dehydrodiferulic acids, hydroxycinnamic acid amides

## Abstract

Maize is an important source of phenolic compounds, specially hydroxycinnamic acids, which are widely known for their antioxidant activity and associated health benefits. However, these effects depend on their bioaccessibility, which is influenced by the different techniques used for food processing. Several traditional products can be obtained from maize and, in Portugal, it is used for the production of an ethnic bread called *broa*. In order to evaluate the effect of processing on maize phenolic composition, one commercial hybrid and five open-pollinated maize flours and *broas* were studied. The total phenolic content and antioxidant activity were evaluated by the Folin-Ciocalteu and ORAC assays, respectively. The major phenolics, namely ferulic and *p*-coumaric acids (in their soluble-free, soluble-conjugated and insoluble forms), insoluble ferulic acid dimers and soluble hydroxycinnamic acid amides were quantitated. Results show that the total phenolic content, antioxidant activity and hydroxycinnamic acids resisted traditional processing conditions used in the production of *broas*. The content in soluble-free phenolics increased after processing, meaning that their bioaccessibility improved. Portuguese traditional *broas*, produced with open-pollinated maize varieties, can be considered an interesting dietary source of antioxidant compounds due to the higher content in hydroxycinnamic acids and derivatives.

## 1. Introduction

Maize (*Zea mays*) is considered a staple cereal in many countries, where it is used to prepare different types of food products [1]. In Portugal, maize is the main ingredient (50–100%) of an ethnic bread known as *broa.* Rye, wheat, (0–50%) or a combination of the flours are also commonly added to the recipe [2]. *Broas* are traditionally prepared using open-pollinated maize varieties. However, Portuguese maize landraces are at risk of disappearing, due to the progressive adoption of more productive hybrid varieties, which originate *broas* less appreciated by consumers [2].

Epidemiological studies have demonstrated that the consumption of phenol-rich foods, as wholegrain cereals, in a regular diet is inversely associated with the risk of developing chronic diseases, such as oncologic and cardiovascular diseases, and metabolic syndrome [3]. Compared to other cereals, maize contains a high level of phenolic compounds, specially hydroxycinnamic acids, which can be found in their soluble or insoluble forms [4]. In particular, the ferulic acid (FA) content is at least ten-fold higher than in other cereal grains, which makes maize one of the most interesting sources of FA in the human diet [4]. Soluble phenolic compounds can be present in their free form or conjugated with smaller molecules, such as simple sugars and amines, as hydroxycinnamic acid amides [5,6,7,8,9]. Insoluble phenolic compounds are mostly (>94%) bound to arabinoxylans [4,5,10], and include dehydrodiferulic, dehydrotriferulic and dehydrotetraferulic acids [4,5] and their derivatives, such as the recently described insoluble hydroxycinnamic acid amides [11].

Evidence from human intervention trials on the protective effects of phenol-rich foods, such as wholegrains, has been inconsistent, mainly due to differences in the food composition and in the bioaccessibility and bioavailability of phenolic compounds [3]. Their bioaccessibility is influenced by the processing techniques involved in the preparation of food products [4,12]. Soluble compounds are usually available for absorption by a simple diffusion mechanism [4,13]. Conversely, insoluble compounds have a very low bioaccessibility [4,13] and need to be liberated from the food matrix during small intestinal digestion or colonic fermentation, in order to be absorbed and become bioavailable [12]. In a previous study, several phenolic compounds, mainly hydroxycinnamic acids and hydroxycinnamic acid amides, were identified in maize flours and *broas* [11].

Since the total content and bioaccessibility of phenolic compounds is influenced by the techniques used for food processing [3], it is mandatory to evaluate the phenolic composition of the final food product. The understanding of the influence of processing also allows the selection of processing conditions that are capable of preserving and increasing the bioaccessibility of phenolic compounds. The study reported herein aimed at elucidating the changes occurring in the soluble-free, soluble-conjugated and insoluble phenolic compositions caused by the processing of raw flours to *broas*, and the potential implications on their bioaccessibility, bioavailability and bioactivity.

## 2. Materials and Methods

### 2.1. Reagents

The reagents and commercial standards used for chromatographic analysis were described elsewhere [11]. Additionally, Folin-Ciocalteu reagent, sodium carbonate, 2′,2′-azobis-(2-amidinopropane) dihydrochloride (AAPH), Trolox and fluorescein sodium salt were obtained from Sigma-Aldrich, St. Louis, MO, USA.

### 2.2. Maize Flour and Broas Preparation

Five traditional Portuguese open-pollinated maize varieties (Broa-213, Pigarro, Castro Verde, Verdeal de Aperrela, Fandango) (Table S1), and their corresponding *broas* were studied. All maize samples were obtained from controlled trials conducted at ESAC (Escola Superior Agrária de Coimbra) and flours were obtained after milling the grain in an artisan water-mill with millstones (Moinhos do Inferno, Viseu, Falling Number 3100). A commercial hybrid maize flour, commonly used for *broa* production (Nacional Type 175) was also included in the study for comparison. This flour was acquired already milled and is described elsewhere [2]. *Broas* were prepared in a bakery following a traditional recipe [14]. The ingredients included 70% maize flour, 20% commercial rye flour (Concordia type 70, Portugal) and 10% commercial wheat flour (National type 65, Portugal). Before extraction of phenolic compounds, *broas* were milled using a grinding mill (IKA MF 10.2, Königswinter, Germany) with a 1.5 mm sieve. The commercial rye and wheat flours used for *broa* production were also analyzed.

### 2.3. Preparation of Phenolic Fractions

**Figure 1:**
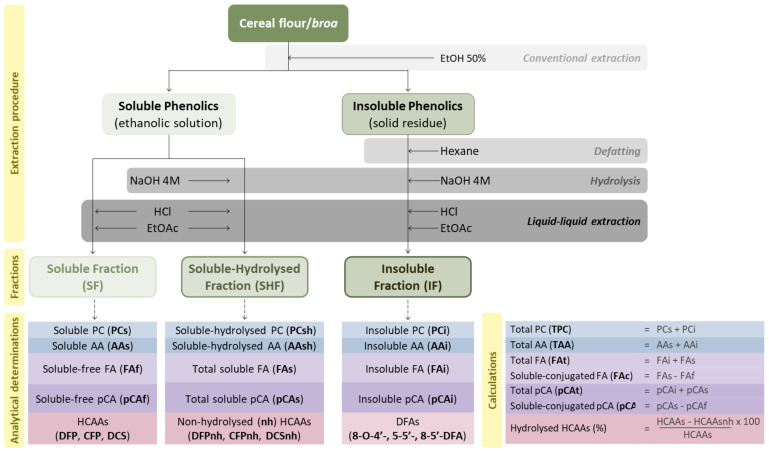
Representative scheme of the soluble (SF), soluble-hydrolyzed (SHF) and insoluble (IF) phenolic fractions and respective determinations.

Cereal flours (maize, rye and wheat) and *broas* (4 g) were submitted to a conventional extraction procedure [11] with 20 mL of EtOH/H_2_O (50%, *v/v*), to obtain an ethanolic solution containing the soluble phenolic compounds and a solid residue comprising the insoluble compounds. Alkaline hydrolyses were applied to both the ethanolic solution and residue. Three phenolic fractions were obtained: the soluble (SF), the soluble-hydrolyzed (SHF) and the insoluble (IF) fractions (Figure 1). The abbreviations used throughout the paper, including tables and figures, are listed in Table 1.

Briefly, in order to obtain the soluble fraction (SF), the pH of the ethanolic solution (5 mL) was adjusted to 1.5 ± 0.5 with concentrated HCl, followed by a liquid-liquid extraction with ethyl acetate (EtOAc, 3 × 7.5 mL). The combined EtOAc fractions were evaporated until dry and reconstituted in 5 mL of EtOH 50%. To obtain the soluble-hydrolyzed fraction (SHF), the ethanolic solution (5 mL) was hydrolyzed with NaOH 4 M (40 mL, pH 14 ± 0.5), under N_2_, for 15 h, at room temperature [15,16]. After hydrolysis, the pH was set to 1.5 ± 0.5 with concentrated HCl, and the phenolic compounds were extracted with EtOAc (3 × 30 mL). The combined EtOAc fractions were evaporated until dry and reconstituted in 5 mL of EtOH 50%.

The insoluble fraction (IF) was prepared from the solid residue, which was defatted with hexane (3 × 20 mL) and centrifuged (7000× *g*, 10 min). The defatted residue was hydrolyzed with NaOH 4 M (60 mL, pH 14 ± 0.5), for 15 h at room temperature, in the presence of N_2_. The obtained solution was extracted with EtOAc (3 × 30 mL), evaporated until dry and reconstituted in 20 mL of EtOH 50%. All fractions (SF, SHF and IF) of cereal flours and *broas* were prepared in duplicate and kept at −20 °C until analysis.

### 2.4. Phenolic Content and Antioxidant Activity

The phenolic content (PC) and antioxidant activity (AA) were determined in each fraction (Figure 1), using Folin-Ciocalteu and ORAC (oxygen radical absorbance capacity) assays, respectively, as previously reported [17]. Determinations were performed in triplicate and reported as mg GAE (gallic acid equivalents) and mmol of Trolox equivalents (TE) per 100 g of sample’s dry weight (dw), respectively. The total PC and the total AA were calculated as described in Figure 1.

### 2.5. HPLC-DAD-ED Analysis

Phenolic compounds were analyzed in a Thermo Fisher Scientific (Waltham, MS, USA) Surveyor high-performance liquid chromatography (HPLC) system, equipped with a diode array detector (DAD) programmed for scanning between 192 and 798 nm and an electrochemical detector, ED 40 Dionex (Sunnyvale, CA, USA). Analytical conditions for HPLC-DAD were previously reported [11]. In order to detect the phenolic compounds that could exhibit AA, electrochemical detection was programmed for a linear variation from −1.0 to 1.0 V in 1.00 s (detection by integrated voltammetry using a cyclic variation of the potential). The measurements were taken with a 50 Hz frequency with an analogic/digital converter.

Hydroxycinnamic acids (FA and pCA) were quantitated in all fractions (SF, SHF and IF) using standard ethanolic solutions prepared from the corresponding commercial standards, at 320 nm, in a range from 0.15 to 100 mg L^−1^ (*y* = 443378*x* + 1415.4, *R*^2^ = 0.9989) and from 0.3 to 50 mg L^−^^1^ (*y* = 624015*x* − 6094.7, *R*^2^ = 0.9998), respectively. The major phenolic compounds (dehydrodiferulic acids and hydroxycinnamic acid amides) were previously identified by HPLC-DAD-MS/MS [11]. Due to the absence of commercially available standards, dehydrodiferulic acids, feruloyl putrescine and coumaroyl feruloyl putrescine were quantitated as FA equivalents (FAE) and dicoumaroyl spermidine was quantitated as pCA equivalents (pCAE). Concentrations were expressed as mg 100 g^−1^ dw.

Limits of quantitation and detection (LOQ and LOD, signal to noise ratio (S/N) of 10 and 3, respectively) of FA and pCA were determined and confirmed by analyzing five independent solutions corresponding to these concentrations. The LOQ for both compounds corresponded to 0.05 mg L^−1^ (approximately 0.03 mg 100 g^−1^ dw) and the LOD to 0.02 mg L^−1^ (0.01 mg 100 g^−1^ dw). In order to control the signal of both detectors (DAD and ED), standard mixtures of FA and pCA at 20 mg L^−1^ were analyzed after every fifteen injections. The total and soluble-conjugated pCA and FA contents were calculated as described in Figure 1.

### 2.6. Data Analysis

ChromQuest (version 3.1.6) software, Thermo Fisher Scientific, Waltham, MA, USA and 4880 software (Unicam, Lisbon, Portugal) were used for data acquisition and treatment of HPLC-DAD and ED analyses, respectively. The identification of pCA and FA was performed by comparison with standard solutions using commercially available standards and the identification of the main dehydrodiferulic acids and hydroxycinnamic acid amides was based on results previously reported [11]. For quantitative data analyses, the limit of significance was set at *p* < 0.05. Paired-samples *t*-tests, independent-samples *t*-tests, ANOVA followed by post hoc Tukey tests, principal component analyses (PCA) and Pearson’s coefficient correlations were obtained using the software SPSS version 21 (IBM, NY, USA).

## 3. Results and Discussion

Maize-based foods can be considered important sources of phenolic compounds in a balanced diet. *Broas* are usually prepared using open-pollinated traditional maize varieties but, more recently, hybrid maize varieties have allowed their production on a larger scale. This work intended to study the total (soluble and insoluble) phenolic composition of five Portuguese traditional maize varieties, which were cultivated in the same environment and in the same period of time, and the corresponding *broas*, contributing to a better understanding of the effect of processing on their bioaccessibility. A commercial maize flour and *broa* were also studied for comparison. As rye and wheat flours were used in *broas* recipes, these flours were also characterized. To achieve these goals, for each cereal flour and *broa* sample, three phenolic fractions were prepared according to Figure 1, namely, the soluble (SF), the soluble-hydrolyzed (SHF) and the insoluble (IF) fractions. The abbreviations used throughout the paper, including tables and figures, are listed in Table 1.

### 3.1. Phenolic Content and Antioxidant Activity of Cereal Flours

The results from the phenolic characterization, phenolic content (PC) and antioxidant activity (AA) of the different cereal flours used in the production of *broas* are presented in Table 2. 

The total PC of maize flours ranged from 150 to 276 mg GAE 100 g^−1^ dw. These values are according to those already described for maize [18,19,20,21,22] but, as expected, are lower than the values reported for pigmented (red, purple, blue or black) varieties (up to 3400 mg GAE 100 g^−1^ dw in purple maize), due to the absence of anthocyanins and other flavonoids [19,23]. For the total AA, values ranged from 3.39 to 6.39 mmol TE 100 g^−1^ dw, which are within the range of the values described in the literature [20,21,23]. The insoluble compounds were responsible for the majority of maize flours’ PC and AA (82.5 ± 3.9% and 85.3 ± 3.3%, respectively) (Table 2).

A strong and positive correlation was observed between insoluble AA and PC (*R* = 0.902, *p* < 0.05) and between soluble AA and PC (*R* = 0.863, *p* < 0.05) (Table 3). These results were expected, as the PC has been described as one of the most important contributors to the AA of cereal grains [23].

The hydrolysis of the SF is usually performed to quantitate the total soluble and conjugated major phenolic acids of maize [18,24,25]. In the present work, the AA and PC were also measured in the SHF (Table 2), in order to evaluate the effect of the hydrolysis procedure on the soluble phenolics and associated AA. The hydrolysis of maize soluble phenolics (from SF to SHF) caused an increase (115 ± 45%) in their AA (*t* = 16.5, *p* < 0.001) (Table S2), suggesting that the hydrolyzed phenolic compounds exhibited a higher AA. Similarly, the PC increased by around 16 ± 7% (*t* = 6.96, *p* < 0.001) after hydrolysis (Table S2), suggesting that this procedure influenced the content of interfering compounds, such as sugars, tyrosine or ascorbic acid, which can influence the PC determined by the Folin-Ciocalteu assay [26]. These findings show the importance of choosing other methods of analysis which can complement the global methods and give more detailed information, enabling the identification and quantitation of individual phenolics. Therefore, HPLC-DAD-ED analyses were performed in all fractions, allowing the quantitation of soluble-free, soluble-conjugated and insoluble ferulic (FA) and *p*-coumaric (pCA) acids and insoluble dehydrodiferulic acids (Figure 1), as previously described for other cereal-based foods [18,24,25]. Additionally, since free and conjugated phenolics may exhibit different antioxidant activities and health effects [7,10], the major soluble-conjugated hydroxycinnamic acids derivatives (hydroxycinnamic acid amides), which are readily available for absorption after consumption, were also quantitated (Figure 1). Moreover, the HPLC-ED analysis allowed the detection of compounds that can have antioxidant potential due to their free radical scavenger ability [27,28]. These results were compared with the AA obtained by the ORAC assay, which evaluates the AA against peroxyl radicals [17].

The results obtained for maize flours (Table 2) show that the total FA and pCA contents ranged from 75.1 to 157.4 and from 4.86 to 14.14 mg 100 g^−1^ dw, respectively. On average, 94.9% of total FA and 84.3% of total pCA of maize flours were present in their insoluble form. Only around 0.2% of the total FA and 1.3% of total pCA were in their soluble-free forms, while 4.9% of total FA and 14.4% of total pCA were soluble, but conjugated with other compounds (soluble-conjugated) (Table 2). These results are also according to the literature [4,18,19,20,21,22,23,29,30,31].

FA and pCA are known to have antioxidant characteristics [27,28] and were detected, as expected, by HPLC-ED analysis. Additionally, maize FA content strongly influenced the AA determined by the ORAC assay, since strong positive correlations were found between AA and FA in both SF and IF (*R* > 0.8, *p* < 0.05) (Table 3). Insoluble FA was responsible for 43.6 ± 4.9% of maize insoluble AA (Table S3). Contrastingly, only around 0.6 ± 0.1% of soluble-free FA and 0.4 ± 0.1% of soluble-free pCA were responsible for maize soluble AA (Table S3). Thus, soluble-free phenolics were not the main contributors of maize soluble AA. Instead, hydroxycinnamic acid amides, which include diferuloyl putrescine, coumaroyl feruloyl putrescine and dicoumaroyl spermidine, the major soluble phenolic compounds present in maize flours (Table 2), can be the main contributors to maize AA.

The *trans*-unsaturated compounds can isomerize into the *cis* form by daylight or during the extraction procedure [4]. As previously reported [11], all the isomeric forms (*cis/cis*, *cis/trans* and *trans/trans*) of diferuloyl putrescine, coumaroyl feruloyl putrescine and dicoumaroyl spermidine were detected in the SF of maize, but the *cis* isomers were only present in trace amounts, below the LOQ of the analytical method (0.03 mg FA/pCA 100 g^−1^ dw). Therefore, in this work, hydroxycinnamic acid amides were quantitated considering the sum of all corresponding isomeric forms which were above the LOQ.

The SHF showed higher (*p* < 0.01) amounts of FA and pCA and lower (*p* < 0.01) amounts of hydroxycinnamic acid amides than the SF (Table 2), which indicated that FA and pCA were released from hydroxycinnamic acid amides during the hydrolysis procedure of the soluble phenolics (Figure 1). The higher amounts in free phenolic acids can also explain the higher AA values obtained for the SHF, suggesting that free FA and pCA are more efficient than hydroxycinnamic acid amides in inhibiting the oxidation induced by peroxyl radicals [17]. Nevertheless, dicoumaroyl spermidine, the most abundant conjugated form of pCA, also contributed for the soluble AA, since very strong and positive correlations were found between the soluble AA with both conjugated pCA (*R* = 0.939, *p* < 0.01) and dicoumaroyl spermidine (*R* = 0.846, *p* < 0.05) (Table 3). Additionally, the ED analysis showed that diferuloyl putrescine exhibited an antioxidant radical-scavenging activity linked to their hydrogen- or electron-donating ability [27,28]. Indeed, it has been reported that hydroxycinnamic acid amides are also potent antioxidants and that feruloyl derivatives exhibited higher radical scavenging activities linked to their hydrogen- or electron-donating ability than soluble-free FA [7].

Maize flours’ SHF still presented considerable amounts of hydroxycinnamic acid amides (Table 2), since only around 28 ± 9% of diferuloyl putrescine, 38 ± 15% of coumaroyl feruloyl putrescine and 76 ± 9% of dicoumaroyl spermidine were hydrolyzed (Table 2). As it has been reported [32], hydroxycinnamic acid amides are difficult to extract quantitatively. Therefore, it is possible that the contents of maize soluble-conjugated FA and pCA have been underestimated.

Other abundant phenolic compounds detected in maize were dehydrodiferulic acids, such as 8-*O*-4′-dehydrodiferulic acid, followed by 5-5′- and 8-5′-dehydrodiferulic acids (Table 2), which were only detected in maize IF, since they are bound to arabinoxylans [4,5,10]. The most abundant dehydrodiferulic acid (8-*O*-4′-) was electrochemically active and therefore showed AA linked to their hydrogen- or electron-donating ability. Additionally, all three main dehydrodiferulic acids showed very strong and positive correlations with the insoluble AA determined by the ORAC assay (*R* > 0.88, *p* < 0.05) and insoluble PC (*R* > 0.95, *p* < 0.01) (Table 3), which suggests they can be important contributors for maize AA against peroxyl radicals and PC. It has also been reported that dehydrodiferulic acids show higher radical-scavenging efficacies than FA [33].

Very strong and positive correlations were also found among maize insoluble FA and the three main dehydrodiferulic acids (*R* > 0.87, *p* < 0.05), between dicoumaroyl spermidine and coumaroyl feruloyl putrescine (*R* = 0.822, *p* < 0.05) and coumaroyl feruloyl putrescine and diferuloyl putrescine (*R* = 0.966, *p* < 0.01) (Table 3). A possible explanation for these correlations could be the different levels of biotic and abiotic stresses that the plants had been exposed, such as drought or salt stress, which are known to increase the content in phenolic acids [4,34]. However, the commercial maize was the only variety which had been exposed to a different environment, and therefore differences in maize genotypes may be the main responsible for the results observed. Indeed, when considering only the traditional samples (*n* = 5, data not shown), which had been submitted to the same edaphoclimatic and agronomic conditions, similar correlations were found, namely, between diferuloyl putrescine and coumaroyl feruloyl putrescine (*R* = 0.927, *p* < 0.05), 5-5′-dehydrodiferulic acid and dicoumaroyl spermidine (*R* = 0.982, *p* < 0.01), 8-*O*-4′- and 5-5′- dehydrodiferulic acids (*R* = 0.966, *p* < 0.01), insoluble FA and 8-*O*-4′-dehydrodiferulic acid (*R* = 0.994, *p* < 0.01) and insoluble FA and 5-5′-dehydrodiferulic acid (*R* = 0.953, *p* < 0.05). Therefore, maize samples with higher hydroxycinnamic acid amides, ferulic acid and dehydrodiferulic acids contents may indicate a higher genetic resistance or tolerance of the variety to biotic and abiotic stresses. Recently, Butts-Wilmsmeyer et al. (2020) [35] showed that, in maize, hydroxycinnamic acids contents are quantitative traits and may be influenced by the environment in which the plants have grown. Furthermore, dehydrodiferulic acids and hydroxycinnamic acid amides can both decrease pathogen penetration into plant tissues [4,36].

As expected [4], when compared to maize, the rye and wheat flours used in *broa* recipes presented lower total PC, total AA and individual phenolics (*p* < 0.01). Moreover, the contribution of the IF for the total PC and total AA was also smaller (*p* < 0.01), with values between 59.6 and 68.1% (Table 2).

### 3.2. Phenolic Content and Antioxidant Activity of Broas

The characterization of *broas* is presented in Table 4. Their total PC (159–223 mg GAE 100 g^−1^ dw) was similar to that reported for other maize-based food products, such as tortillas and tortilla chips (60.7–207 mg 100 g^−1^ dw) [18,21]. In particular, the FA and pCA contents obtained for *broas* (81.6 ± 10.1 and 7.4 ± 2.8 mg 100 g^−1^ dw, respectively) were higher than the contents described for rye (54.0 and 2.8 mg 100 g^−1^) and wheat (8.2 and 0.28 mg 100 g^−1^) breads [37], possibly due to the higher phenolic contents of maize [4].

In *broas*, the IF was responsible for 71.6 ± 3.0% of total PC and 77.9 ± 1.9% of the total AA (Table 4). On average, 95.9% of total FA and 81.3% of total pCA of *broas* were present in their insoluble form. Only around 0.99% and 8.2% of the total FA and pCA, respectively, were in their soluble-free forms, whilst 3.7% of total FA and 10.9% of total pCA were soluble, but conjugated with other compounds (Table 4).

As for maize flours, hydroxycinnamic acid amides and dehydrodiferulic acids were detected as major phenolic compounds present in *broas* SF and IF, respectively (Table 4). Differences in the maize varieties used for *broas* production can explain the strong and positive correlations among the insoluble FA and dehydrodiferulic acids (*R* > 0.85, *p* < 0.05), as well as between dicoumaroyl spermidine and dehydrodiferulic acids (*R* > 0.92, *p* < 0.05) (Table 5).

Contrary to the observations for maize flours, the hydrolysis of *broa* soluble phenolics (SF) (Figure 1) did not influence its soluble PC (*p* > 0.05) (Table S4), which may indicate the presence of less interfering compounds. Nevertheless, the hydrolysis procedure also caused an average increase of 45 ± 28% in *broas* soluble AA (Table S4) (*t* = 6.3, *p* < 0.001), which was lower than the increase observed for maize flours (115 ± 45%). As previously discussed, this increase can be explained by higher free FA and pCA contents in the SHF. These findings imply that, in *broas*, the amount of FA and pCA released after hydrolysis was lower than in maize flours, due to lower contents in soluble-conjugated phenolics. Indeed, the contribution of compounds other than FA and pCA for the soluble AA of maize flours was around 99.0% (Table S3), while in *broas* it was around 96.9% (Table S5) (*t* = 14.4, *p* < 0.01).

Soluble-free FA and pCA and dicoumaroyl spermidine contents strongly influenced *broas* soluble AA (*R* > 0.86, *p* < 0.05), while insoluble pCA and 8-5′-dehydrodiferulic acid strongly influenced *broas* insoluble AA (R > 0.81, *p* < 0.05) (Table 4). Since hydroxycinnamic acid amides and dehydrodiferulic acids were detected in *broas* as abundant phenolic compounds with antioxidant properties, they may contribute to *broas* health promoting effects. After consumption, dehydrodiferulic acids can be released from the matrix during digestion and further absorbed [38]. To the best of our knowledge, there is no data on hydroxycinnamic acid amides bioavailability, but it is known that other FA conjugates, such as feruloylated oligosaccharides, can be absorbed by a diffusion mechanism [4]. Additionally, some insoluble phenolics may also exhibit their beneficial action directly in the gastrointestinal system [4].

### 3.3. Traditional and Commercial Maize Flours and Broas Comparison

**Figure 2:**
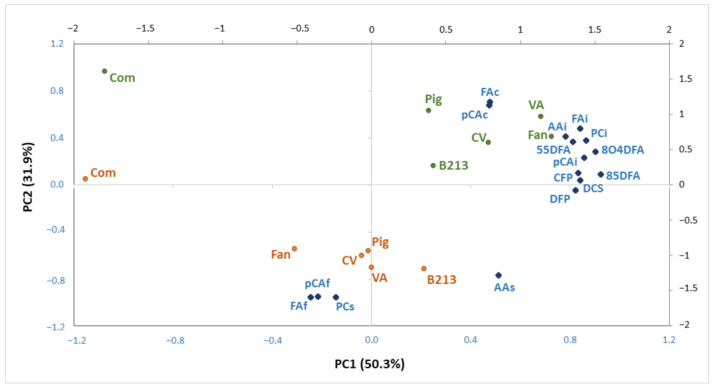
Projection of maize flours (green), *broas* (brown) and variables (blue) (Table 1) in the plane defined by PC1 and PC2, corresponding to 82.2% of total variance.

In order to study the main differences among maize samples varieties and corresponding *broas*, a principal component analysis (PCA) was performed using 16 variables, described in Table 2 and Table 4. Figure 2 shows the projection of samples and variables in the space defined by the two principal components, corresponding to 82.2% of the total variance. Three sample clusters can be identified: (1) traditional maize flours, (2) traditional *broas* and (3) commercial maize flour and the corresponding *broa*, which are projected along different directions.

The commercial maize flour and corresponding *broa* were differentiated from all the other samples along PC1, mainly due to the lower contents of insoluble phenolics and hydroxycinnamic acid amides, as described in Table 1 and Table 3. Indeed, post hoc analysis revealed that the contents in hydroxycinnamic acid amides, 8-*O*-4′- and 8-5′-dehydrodiferulic acids of the commercial *broa* were significantly lower (*p* < 0.05) than all the traditional *broas* (Table 4). Similar results were observed for its soluble and total AA (Table 4). These differences can be explained by the different genotype and variety type, since it was a hybrid, whereas the traditional samples were open-pollinated varieties [25]; and also by its different edaphoclimatic and agronomic growing conditions [20,23]. Moreover, as previously reported, the commercial maize flour used in the present work presented a higher mean diameter and large particle distribution than the traditional flours [2]. This could have influenced not only the extraction of maize phenolic compounds, but also the amount of phenolic compounds released during the breadmaking process [13] and, consequently, the corresponding *broa* AA. As previously reported [2], a sensory evaluation study has demonstrated a preference for traditional in detriment of hybrid maize varieties for *broa* production, suggesting that, even without previous knowledge, consumers prefer *broas* with higher contents in these health promoting compounds.

*Broas* were discriminated from maize flours along PC2, mainly due to their higher contents of soluble PC and AA, as well as soluble-free FA and pCA, and by lower contents of soluble-conjugated FA and pCA. These results suggest that the content of soluble-free phenolics increased as a consequence of maize processing to *broas*, as discussed below.

### 3.4. From Raw Flours to Broas

All *broas* (*n* = 6) were prepared following the same recipe, which included not only maize flour, but also 20% rye and 10% wheat flours. Therefore, the values obtained for the raw flours’ mixture (RF) that was used for the preparation of *broas* were calculated (Table S6) according to the expression:

RF = 0.70 × M + 0.20 × *R* + 0.10 × W, (1)

where M, R and W are the values presented in Table 2 for the different varieties of maize (*n* = 6) and for the rye and wheat flours, respectively. Results from raw flours and *broas* were compared based on the formula:

100 × B/RF,(2)

which enabled the determination of the amount of AA, PC and individual phenolics remaining after raw flour (RF) processing to *broas* (B) (Table 6).

A paired-samples *t*-test was performed to compare the AA and phenolic composition of raw flours and the corresponding *broas*. The results presented in Table 6 show that the soluble PC and AA significantly increased in *broas*, possibly due to the increase (≥three-fold) in the soluble-free FA and pCA contents. Similar results have been obtained for other maize products, particularly for tortillas, tortilla chips and cornflakes [1,18,20,31], as well as for other breads [25,39]. Some authors argued that the increase in soluble-free phenolics might be caused by the release of insoluble FA and pCA that occurs during breadmaking, especially during the fermentation process [13,18,24,25,39,40]. Therefore, a decrease in the insoluble phenolic content should be expected after processing. However, no significant differences (*p* > 0.05) were found concerning insoluble PC, AA, FA, pCA or dehydrodiferulic acids contents (Table 6). Different hypotheses can explain these results. Firstly, the amount of FA and pCA released during the processing corresponded only to 0.6–1.1% and 4.6–9.9% of the total FA and pCA contents originally present in the raw flours, corroborating the results described for rye processing to bread [39]. Secondly, a considerable amount of insoluble phenolics can remain linked to cell walls even after hydrolysis [4]. Ultimately, the increase in soluble-free phenolics might have been caused by the hydrolysis of soluble-conjugated phenolic compounds, in particular hydroxycinnamic acid amides [25]. Indeed, after processing, the soluble-conjugated FA and pCA contents decreased by around 27 and 44%, respectively (*p* < 0.05) (Table 6).

Since the soluble-conjugated FA and pCA contents decreased after processing, a decrease in the content of the main soluble-conjugated phenolics (hydroxycinnamic acid amides) would be expected. However, no differences were found between raw flours and *broas* regarding their content in diferuloyl putrescine, coumaroyl feruloyl putrescine or dicoumaroyl spermidine (Table 6). This suggests that insoluble hydroxycinnamic acid amides, recently described in maize flours and *broas* [11], may have been released during processing, therefore increasing the content of soluble hydroxycinnamic acid amides and soluble-free FA and pCA. Additionally, other minor soluble-conjugated compounds, namely, hydroxycinnamic acid amides monoconjugates, may have been hydrolyzed during processing, contributing to the higher soluble-free and lower soluble-conjugated FA and pCA contents detected in *broas*.

**Figure 3:**
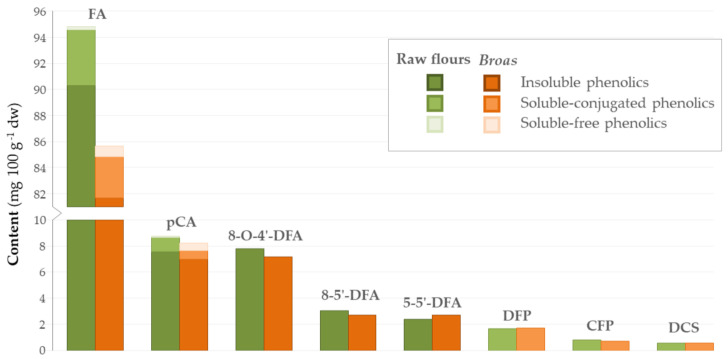
Average contents of phenolic compounds obtained for the traditional raw flours (green, *n* = 5) and *broas* (brown, *n* = 5).

A comparison of traditional raw flours and *broas’* main phenolic compounds is presented in Figure 3. No significant differences (*p* > 0.05) were found between raw flours and *broas* regarding their total FA, pCA, hydroxycinnamic acid amides and dehydrodiferulic acids contents (Table 6). Contradictory results have been reported regarding the processing effects on the phenolic composition of other cereals, such as wheat and rye, to breads. Some authors concluded that the processing did not influence the content in the major phenolics, including FA and pCA [37]. On the other hand, some authors reported that the content of total phenolics and total FA, in particular, decreased after processing [39], while others have shown that it increased [25,41]. These discrepancies can be explained by differences in sample genotypes [13,25,39] and breadmaking processing conditions, namely, in dough pH, which influences the mechanical disaggregation of cell walls and the acidic hydrolysis that may occur during mixing [13,25,39]. No differences have been found between the dehydrodiferulic acids contents of rye flours and breads [25,39], which is in accordance to the present findings. To the best of our knowledge, hydroxycinnamic acid amides have not been considered in previous studies.

On the other hand, after maize grain processing into tortillas, there is a 56–90% reduction in the total PC, mainly due to leaching of insoluble FA during the nixtamalization process [18,21,42]. The content of phenolic acids also decreased after processing of maize into toasted cornflakes due to the pressure cooking stage and to the dry milling process, which involved the removal of maize bran and germ [31]. Therefore, the results obtained in the present work suggest that, in comparison to other maize-based foods, *broas* are an interesting source of phenolic compounds, since there were no significant losses in the total phenolics caused by the processing conditions, which were based on a traditional *broa* recipe.

## 4. Conclusions

*Broa*, a Portuguese traditional maize bread, can be considered an interesting source of antioxidant compounds, particularly hydroxycinnamic acids and hydroxycinnamic acid amides. *Broas* produced with Portuguese traditional open-pollinated maize varieties, which were, in a previous work, associated with better sensory characteristics, also showed higher phenolic content than the *broa* prepared with a commercial maize flour. The processing conditions used in *broa* preparation did not significantly change the phenolic content present in the raw flours used for its production. There was an increase in soluble-free phenolics after processing, suggesting that phenolic compounds’ bioaccessibility was improved in *broas*. In vitro studies are currently being carried out to understand which compounds are bioavailable and may contribute to considering *broa* as a bread with health-promoting properties.

## Figures and Tables

**Table 1:** List of abbreviations.

Abbreviation	Definition	Abbreviation	Definition
55DFA	5-5′-Dehydrodiferulic acid	Fan	Fandango
85DFA	8-5′-Dehydrodiferulic acid	GAE	Gallic acid equivalents
8O4DFA	8-O-4′-Dehydrodiferulic acid	HCAAs	Hydroxycinnamic acid amides
AA	Antioxidant activity	HPLC	High performance liquid chromatography
AAi	Insoluble antioxidant activity	IF	Insoluble fraction
AAs	Soluble antioxidant activity	LOD	Limit of detection
B213	*Broa* 213	LOQ	Limit of quantification
CFP	Coumaroyl feruloyl putrescine	n/a	Not applicable
Com	Commercial	ORAC	Oxygen radical absorbance capacity
CV	Castro Verde	PC	Phenolic content
DAD	Diode array detector	PCi	Insoluble phenolic content
DCS	Dicoumaroyl spermidine	PCs	Soluble phenolic content
DFAs	Dehydrodiferulic acids	pCA	*p*-Coumaric acid
DFP	Diferuloyl putrescine	pCAc	Soluble-conjugated *p*-coumaric acid
dw	Dry weight	pCAf	Soluble-free *p*-coumaric acid
ED	Electrochemical detector	pCAi	Insoluble *p*-coumaric acid
EtOAC	Ethyl acetate	PCA	Principal component analysis
EtOH	Ethanol	Pig	Pigarro
FA	Ferulic acid	SF	Soluble fraction
FAc	Soluble-conjugated ferulic acid	SHF	Soluble-hydrolyzed fraction
FAE	Ferulic acid equivalents	TE	Trolox equivalents
FAf	Soluble-free ferulic acid	VA	Verdeal de Aperrela
FAi	Insoluble ferulic acid		

**Table 2:** Cereal flours mean values for: phenolic content, antioxidant activity and individual phenolic contents (ferulic acid, *p*-coumaric acid, diferuloyl putrescine, coumaroyl feruloyl putrescine, dicoumaroyl spermidine and dehydrodiferulic acids) in the different fractions, as described in Figure 1. Mean values within rows with no letters (a–d) in common are significantly different (*p* < 0.05).

Description	Phenolic Fraction	Maize							Wheat	Rye
		Broa-213	Pigarro	Castro Verde	Verdeal de Aperrela	Fandango	Commercial	Average		
		**Phenolic Content (PC)** (mg GAE 100 g^−1^ dw)								
Soluble	SF	47.9 ± 2.3 ^a^	37.0 ± 0.9 ^ab^	37.9 ± 5.1 ^ab^	41.7 ± 2.0 ^ab^	37.1 ± 2.5 ^ab^	32.2 ± 3.1 ^b^	39.0 ± 5.3	5.73 ± 0.1	15.9 ± 0.9
Soluble-hydrolyzed	SHF	52.2 ± 1.9 ^ab^	42.6 ± 1.4 ^ab^	42.6 ± 4.9 ^ab^	53.0 ± 3.3 ^a^	41.0 ± 2.2 ^ab^	39.2 ± 3.0 ^b^	45.1 ± 6.0	6.87 ± 0.5	17.1 ± 1.0
Insoluble	IF	157 ± 8.4 ^ab^	200 ± 19.4 ^ac^	203 ± 9.25 ^ac^	226 ± 22.7 ^ac^	239 ± 17.6 ^c^	118 ± 9.0 ^b^	190 ± 45.3	12.3 ± 1.0	27.6 ± 0.7
Total	SF + IF	205 ± 10.7 ^ab^	237 ± 20.3 ^ac^	240 ± 14.4 ^ac^	268 ± 24.7 ^ac^	276 ± 20.1 ^c^	150 ± 12.1 ^b^	229 ± 46.4	18.0 ± 1.1	43.5 ± 1.6
% Insoluble	n/a	76.6	84.4	84.3	84.5	86.6	78.5	82.5 ± 3.9	68.1	63.4
		**Antioxidant Activity (AA)** (mmol TE 100 g^−1^ dw)								
Soluble	SF	0.87 ± 0.05 ^a^	0.75 ± 0.11 ^a^	0.75 ± 0.18 ^a^	0.72 ± 0.08 ^a^	0.66 ± 0.07 ^a^	0.46 ± 0.04 ^a^	0.70 ± 0.14	0.17 ± 0.02	0.42 ± 0.03
Soluble-hydrolyzed	SHF	1.65 ± 0.08 ^a^	1.59 ± 0.20 ^a^	1.28 ± 0.20 ^a^	1.39 ± 0.33 ^a^	1.51 ± 0.15 ^a^	1.38 ± 0.11 ^a^	1.47 ± 0.14	0.13 ± 0.02	0.47 ± 0.07
Insoluble	IF	3.40 ± 0.24 ^ab^	4.66 ± 0.71 ^ab^	4.02 ± 0.44 ^ab^	4.38 ± 0.73 ^ab^	5.73 ± 0.80 ^a^	2.93 ± 0.48 ^b^	4.19 ± 0.99	0.30 ± 0.02	0.62 ± 0.07
Total	SF + IF	4.27 ± 0.29 ^ab^	5.41 ± 0.83 ^ab^	4.76 ± 0.61 ^ab^	5.10 ± 0.81 ^ab^	6.39 ± 0.88 ^a^	3.39 ± 0.52 ^b^	4.89 ± 1.02	0.47 ± 0.04	1.03 ± 0.10
% Insoluble	n/a	79.6	86.2	84.3	85.8	89.7	86.3	85.3 ± 3.3	63.7	59.6
		**Ferulic acid (FA)** (mg 100 g^−1^ dw) and contribution (%) for the total FA **(bold)**								
Soluble-free	SF	0.35 ± 0.01 ^a^ **(0.3)**	0.24 ± 0.01 ^ab^ **(0.2)**	0.25 ± 0.05 ^ab^ **(0.2)**	0.29 ± 0.06 ^ab^ **(0.2)**	0.23 ± 0.02 ^ab^ **(0.1)**	0.18 ± 0.04 ^b^ **(0.2)**	0.26 ± 0.06 **(0.2)**	0.10 ± 0.01 **(3.0)**	0.26 ± 0.01 **(3.6)**
Soluble-conjugated	SHF-SF	6.49 ± 0.00 ^a^ **(6.3)**	5.80 ± 1.44 ^a^ **(4.5)**	4.57 ± 2.33 ^a^ **(3.6)**	6.66 ± 0.43 ^a^ **(4.5)**	5.21 ± 0.51 ^a^ **(3.3)**	5.32 ± 0.13 ^a^ **(7.1)**	5.67 ± 0.80 **(4.9)**	0.25 ± 0.02 **(7.3)**	1.08 ± 0.00 **(14.9)**
Total soluble	SHF	6.84 ± 0.02 ^a^ **(6.6)**	6.04 ± 1.43 ^a^ **(4.7)**	4.81 ± 2.38 ^a^ **(3.8)**	6.95 ± 0.49 ^a^ **(4.7)**	5.44 ± 0.53 ^a^ **(3.4)**	5.49 ± 0.10 ^a^ **(7.3)**	5.93 ± 0.84 **(5.1)**	0.35 ± 0.03 **(10.3)**	1.34 ± 0.01 **(18.5)**
Insoluble	IF	95.8 ± 14.0 ^ab^ **(93.3)**	122 ± 18.0 ^abc^ **(95.3)**	122 ± 10.0 ^abc^ **(96.2)**	143 ± 21.6 ^bc^ **(95.4)**	152 ± 7.2 ^c^ **(96.5)**	69.6 ± 2.32 ^a^ **(92.7)**	117 ± 30.4 **(94.9)**	3.04 ± 0.24 **(89.7)**	5.92 ± 0.49 **(81.6)**
Total	SHF + IF	103 ± 14.0 ^ab^	128 ± 19.4 ^ab^	127 ± 12.3 ^ab^	150 ± 21.1 ^a^	157 ± 7.8 ^a^	75.1 ± 2.2 ^b^	123 ± 31.5	3.39 ± 0.20	7.26 ± 0.50
		***p*-Coumaric acid (pCA)** (mg 100 g^−1^ dw) and contribution (%) for the total pCA **(bold)**								
Soluble-free	SF	0.16 ± 0.03 ^a^ **(1.5)**	0.09 ± 0.00 ^a^ **(0.6)**	0.15 ± 0.05 ^a^ **(1.2)**	0.11 ± 0.01 ^a^ **(0.9)**	0.12 ± 0.01 ^a^ **(1.1)**	0.12 ± 0.01 ^a^ **(2.5)**	0.13 ± 0.03 **(1.3)**	0.004 ± 0.00 **(4.3)**	0.06 ± 0.02 **(8.7)**
Soluble-conjugated	SHF-SF	1.84 ± 0.10 ^a^ **(16.8)**	1.63 ± 0.32 ^a^ **(11.5)**	1.56 ± 0.63 ^a^ **(12.1)**	1.46 ± 0.06 ^a^ **(12.1)**	1.24 ± 0.04 ^a^ **(10.9)**	1.11 ± 0.04 ^a^ **(22.8)**	1.48 ± 0.27 **(14.4)**	0.01 ± 0.00 **(7.4)**	0.21 ± 0.02 **(27.9)**
Total soluble	SHF	2.00 ± 0.13 ^a^ **(18.3)**	1.72 ± 0.32 ^a^ **(12.1)**	1.72 ± 0.68 ^a^ **(13.3)**	1.58 ± 0.05 ^a^ **(13.0)**	1.37 ± 0.04 ^a^ **(12.0)**	1.23 ± 0.03 ^a^ **(25.3)**	1.60 ± 0.28 **(15.7)**	0.01 ± 0.00 **(11.7)**	0.27 ± 0.00 **(36.6)**
Insoluble	IF	8.96 ± 2.01 ^a^ **(81.7)**	12.41 ± 5.80 ^a^ **(87.8)**	11.2 ± 4.16 ^a^ **(86.7)**	10.5 ± 1.82 ^a^ **(87.0)**	10.0 ± 0.72 ^a^ **(88.0)**	3.63 ± 0.94 ^a^ **(74.7)**	9.45 ± 3.08 **(84.3)**	0.08 ± 0.01 **(88.2)**	0.47 ± 0.03 **(63.4)**
Total	SHF + IF	10.96 ± 2.1 ^a^	14.14 ± 6.1 ^a^	12.90 ± 4.8 ^a^	12.08 ± 1.8 ^a^	11.38 ± 0.8 ^a^	4.86 ± 0.97 ^a^	11.05 ± 3.24	0.09 ± 0.01	0.74 ± 0.02
		**Diferuloyl putrescine (DFP)** (mg FAE 100 g^−1^ dw)								
DFP	SF	2.61 ± 0.15 ^ab^	1.24 ± 0.27 ^ac^	2.73 ± 1.02 ^ab^	3.37 ± 0.13 ^b^	1.95 ± 0.03 ^abc^	0.47 ± 0.02 ^c^	2.06 ± 1.07	<0.03	<0.03
Non-hydrolyzed DFP	SHF	1.76 ± 0.14 ^ab^	0.84 ± 0.12 ^ac^	1.62 ± 0.38 ^ab^	2.74 ± 0.56 ^b^	1.36 ± 0.05 ^ac^	0.39 ± 0.01 ^c^	1.45 ± 0.81	<0.03	<0.03
% Hydrolyzed	n/a	32	32	41	19	30	17	28 ± 9	n/a	n/a
		**Coumaroyl feruloyl putrescine (CFP)** (mg FAE 100 g^−1^ dw)								
CFP	SF	1.40 ± 0.15 ^a^	0.59 ± 0.02 ^bc^	1.11 ± 0.25 ^a^	1.56 ± 0.08 ^a^	1.08 ± 0.03 ^ab^	0.26 ± 0.03 ^c^	1.00 ± 0.49	<0.03	<0.03
Non-hydrolyzed CFP	SHF	0.76 ± 0.07 ^abc^	0.28 ± 0.03 ^ad^	0.51 ± 0.18 ^abd^	1.20 ± 0.15 ^c^	0.87 ± 0.21 ^bc^	0.17 ± 0.00 ^d^	0.63 ± 0.39	<0.03	<0.03
% Hydrolyzed	n/a	46	53	54	23	19	34	38 ± 15	n/a	n/a
		**Dicoumaroyl spermidine (DCS)** (mg pCAE 100 g^−1^ dw)								
DCS	SF	1.01 ± 0.06 ^a^	0.54 ± 0.05 ^ab^	0.93 ± 0.30 ^a^	0.71 ± 0.07 ^ab^	0.79 ± 0.03 ^a^	0.25 ± 0.03 ^b^	0.71 ± 0.28	<0.03	<0.03
Non-hydrolyzed DCS	SHF	0.21 ± 0.00 ^a^	0.14 ± 0.00 ^ab^	0.13 ± 0.01 ^ab^	0.29 ± 0.05 ^a^	0.22 ± 0.08 ^a^	0.04 ± 0.02 ^b^	0.17 ± 0.08	<0.03	<0.03
% Hydrolyzed	n/a	80	74	86	59	73	83	76 ± 9	n/a	n/a
		**Dehydrodiferulic acids (DFA)** (mg FAE 100 g^−1^ dw)								
8-O-4′-DFA	IF	6.81 ± 0.16 ^a^	10.0 ± 5.29 ^a^	10.9 ± 4.63 ^a^	12.89 ± 3.21 ^a^	14.48 ± 0.02 ^a^	4.04 ± 0.99 ^a^	9.85 ± 3.87	0.14 ± 0.01	0.32 ± 0.02
5-5′-DFA	IF	2.43 ± 0.10 ^a^	4.03 ± 2.60 ^a^	4.24 ± 2.07 ^a^	4.59 ± 1.05 ^a^	6.27 ± 0.41 ^a^	1.92 ± 0.45 ^a^	3.92 ± 1.57	0.04 ± 0.01	0.11 ± 0.00
8-5′-DFA	IF	2.39 ± 0.24 ^a^	3.78 ± 1.56 ^a^	3.13 ± 1.37 ^a^	3.66 ± 1.19 ^a^	3.77 ± 0.63 ^a^	1.44 ± 0.45 ^a^	3.03 ± 0.94	0.07 ± 0.00	0.21 ± 0.04

**Table 3:** Correlation coefficients among maize variables, as described in Table 1. Very strong correlations (|*R*| > 0.8) are highlighted in bold. *p*-Value corresponds to the significance level of Pearson correlation coefficient indicated as *: significant at *p* < 0.05; **: significant at *p* < 0.01.

		SF							SHF-SF		IF						
		PCs	AAs	FAf	pCAf	DFP	CFP	DCS	FAc	pCAc	PCi	AAi	FAi	pCAi	8O4DFA	55DFA	85DFA
SF	**PCs**		0.863 *	0.996 **	0.521	0.715	0.813 *	0.776	0.665	0.810	0.137	−0.065	0.146	0.352	0.097	−0.073	0.147
	**AAs**			0.887 *	0.368	0.687	0.716	0.846 *	0.403	0.939 **	0.375	0.185	0.356	0.731	0.306	0.154	0.460
	**FAf**				0.494	0.743	0.821 *	0.779	0.663	0.841 *	0.162	−0.065	0.166	0.404	0.116	−0.066	0.184
	**pCAf**					0.388	0.409	0.664	−0.136	0.369	−0.275	−0.413	−0.283	−0.168	−0.253	−0.295	−0.420
	**DFP**						0.966 **	0.794	0.354	0.506	0.578	0.219	0.572	0.551	0.562	0.371	0.472
	**CFP**							0.822 *	0.472	0.512	0.556	0.262	0.565	0.492	0.547	0.373	0.447
	**DCS**								0.096	0.680	0.451	0.254	0.439	0.575	0.434	0.327	0.376
SHF-SF	**FAc**									0.420	−0.004	−0.120	0.029	0.072	−0.044	−0.203	0.080
	**pCAc**										0.082	−0.102	0.055	0.588	−0.002	−0.159	0.230
IF	**PCi**											0.902 *	0.998 **	0.794	0.994 **	0.950 **	0.952 **
	**AAi**												0.912 *	0.652	0.909 *	0.961 **	0.882 *
	**FAi**													0.762	0.996 **	0.955 **	0.942 **
	**pCAi**														0.729	0.633	0.898 *
	**8O4DFA**															0.970 **	0.918 **
	**55DFA**																0.866 *
	**85DFA**																

**Table 4:** *Broas* mean values for: phenolic content, antioxidant activity and individual phenolic contents (ferulic acid, *p*-coumaric acid, diferuloyl putrescine, coumaroyl feruloyl putrescine, dicoumaroyl spermidine and dehydrodiferulic acids) obtained in the different fractions, as described in Figure 1. Mean values within rows with no letters (a–d) in common (a-dare significantly different (*p* < 0.05).

Description	Phenolic Fraction	Broa-213	Pigarro	Castro Verde	Verdeal de Aperrela	Fandango	Commercial	Average
**Phenolic Content (PC)** (mg GAE 100 g^−1^ dw)								
Soluble	SF	56.9 ± 3.0 ^a^	53.5 ± 2.7 ^a^	53.2 ± 3.7 ^a^	58.1 ± 2.3 ^a^	59.8 ± 1.2 ^a^	52.1 ± 5.6 ^a^	55.6 ± 3.1
Soluble-hydrolyzed	SHF	55.8 ± 1.7 ^a^	62.4 ± 1.9 ^ab^	67.8 ± 2.0 ^b^	69.9 ± 4.8 ^b^	55.6 ± 1.0 ^a^	52.1 ± 1.7 ^a^	60.6 ± 7.2
Insoluble	IF	149 ± 13.6 ^a^	169 ± 4.22 ^a^	144 ± 6.50 ^a^	138 ± 2.69 ^ab^	145 ± 10.5 ^a^	107 ± 2.03 ^b^	142 ± 20.4
Total	SF + IF	206 ± 16.6 ^a^	223 ± 6.92 ^a^	198 ± 10.2 ^ab^	196 ± 5.06 ^ab^	205 ± 11.8 ^a^	159 ± 7.60 ^b^	197 ± 21.3
% Insoluble	n/a	72.3	76	73.1	70.3	70.7	67.2	71.6 ± 3.0
**Antioxidant Activity (AA)** (mmol TE 100 g^−1^ dw)								
Soluble	SF	0.99 ± 0.07 ^a^	0.92 ± 0.07 ^ab^	0.86 ± 0.13 ^ab^	0.90 ± 0.18 ^ab^	0.79 ± 0.03 ^b^	0.54 ± 0.03 ^c^	0.83 ± 0.16
Soluble-hydrolyzed	SHF	1.22 ± 0.13 ^a^	1.28 ± 0.12 ^a^	1.39 ± 0.26 ^a^	1.23 ± 0.12 ^a^	0.92 ± 0.19 ^a^	1.04 ± 0.00 ^a^	1.18 ± 0.17
Insoluble	IF	3.12 ± 0.70 ^a^	3.95 ± 0.15 ^a^	2.82 ± 0.53 ^ab^	3.20 ± 0.16 ^a^	2.94 ± 0.13 ^ab^	1.76 ± 0.26 ^b^	2.97 ± 0.71
Total	SF + IF	4.11 ± 0.77 ^a^	4.87 ± 0.22 ^a^	3.68 ± 0.65 ^a^	4.10 ± 0.35 ^a^	3.73 ± 0.16 ^a^	2.30 ± 0.29 ^b^	3.80 ± 0.85
% Insoluble	n/a	76	81.2	76.7	78.1	78.9	76.4	77.9 ± 1.9
**Ferulic acid (FA)** (mg 100 g^−1^ dw) and contribution (%) for the total FA **(bold)**								
Soluble-free	SF	0.91 ± 0.14 ^a^ **(0.97)**	0.80 ± 0.24 ^a^ **(0.93)**	0.78 ± 0.00 ^a^ **(0.94)**	0.81 ± 0.06 ^a^ **(0.95)**	0.79 ± 0.18 ^a^ **(1.03)**	0.71 ± 0.11 ^a^ **(1.10)**	0.80 ± 0.07 **(0.99)**
Soluble-conjugated	SHF-SF	4.31 ± 0.45 ^a^ **(4.6)**	2.43 ± 0.15^a^ **(2.9)**	3.30 ± 1.09 ^a^ **(4.0)**	2.87 ± 1.34 ^a^ **(3.4)**	2.95 ± 0.18 ^a^ **(3.8)**	2.47 ± 0.42 ^a^ **(3.8)**	3.05 ± 0.69 **(3.7)**
Total soluble	SHF	5.22 ± 0.31 ^a^ **(5.3)**	3.23 ± 0.08 ^a^ **(3.8)**	4.08 ± 1.09 ^a^ **(4.9)**	3.68 ± 1.40 ^a^ **(4.4)**	3.87 ± 0.00 ^a^ **(4.8)**	3.18 ± 0.53 ^a^ **(4.9)**	3.88 ± 0.75 **(4.7)**
Insoluble	IF	89.3 ± 0.1 ^a^ **(94.4)**	82.1 ± 15.5 ^a^ **(96.2)**	79.3 ± 0.59 ^a^ **(95.1)**	81.8 ± 0.63 ^a^ **(95.7)**	75.9 ± 4.18 ^a^ **(95.2)**	61.4 ± 18.2 ^a^ **(95.1)**	78.27 ± 9.4 **(95.9)**
Total	SHF + IF	94.5 ± 2.62 ^a^	85.3 ± 15.38 ^a^	83.3 ± 0.51 ^a^	85.5 ± 0.78 ^a^	79.8 ± 1.95 ^a^	64.5 ± 17.69 ^a^	81.62 ± 10.06
***p*-Coumaric acid (pCA)** (mg 100 g^−1^ dw) and contribution (%) for the total pCA **(bold)**								
Soluble-free	SF	0.66 ± 0.03 ^a^ **(7.4)**	0.57 ± 0.09 ^a^ **(5.3)**	0.63 ± 0.01 ^a^ **(6.9)**	0.54 ± 0.03 ^a^ **(7.6)**	0.53 ± 0.17 ^a^ **(10.4)**	0.39 ± 0.03 ^a^ **(11.7)**	0.55 ± 0.09 **(8.2)**
Soluble-conjugated	SHF-SF	1.06 ± 0.09 ^a^ **(11.8)**	0.53 ± 0.00 ^a^ **(4.9)**	0.82 ± 0.33 ^a^ **(9.0)**	0.59 ± 0.34 ^a^ **(8.4)**	0.43 ± 0.23 ^a^ **(8.5)**	0.78 ± 0.10 ^a^ **(23.1)**	0.70 ± 0.23 **(10.9)**
Total soluble	SHF	1.72 ± 0.06 ^a^ **(19.2)**	1.10 ± 0.09 ^a^ **(10.2)**	1.45 ± 0.34 ^a^ **(15.9)**	1.13 ± 0.31 ^a^ **(16.0)**	1.08 ± 0.20 ^a^ **(18.9)**	1.17 ± 0.13 ^a^ **(34.8)**	1.28 ± 0.26 **(19.2)**
Insoluble	IF	7.30 ± 0.1 ^a^ **(80.8)**	9.71 ± 1.4 ^a^ **(89.8)**	7.66 ± 0.6 ^ab^ **(84.1)**	5.92 ± 0.0 ^bc^ **(83.9)**	4.23 ± 0.3 ^cd^ **(81.1)**	2.20 ± 0.48 ^d^ **(65.2)**	6.17 ± 2.67 **(81.3)**
Total	SHF + IF	9.03 ± 0.23 ^ab^	10.80 ± 1.34 ^a^	9.11 ± 0.28 ^ab^	7.05 ± 0.33 ^b^	5.31 ± 0.16 ^bc^	3.36 ± 0.36 ^c^	7.40 ± 2.79
**Diferuloyl putrescine (DFP)** (mg FAE 100 g^−1^ dw)								
DFP	SF	1.76 ± 0.07 ^a^	1.14 ± 0.31 ^a^	1.70 ± 0.15 ^a^	2.52 ± 0.05 ^b^	1.34 ± 0.15 ^a^	0.38 ± 0.05 ^c^	1.47 ± 0.71
Non-hydrolyzed DFP	SHF	1.72 ± 0.05 ^ab^	0.75 ± 0.08 ^c^	1.56 ± 0.17 ^ab^	1.87 ± 0.46 ^a^	0.99 ± 0.01 ^bc^	0.33 ± 0.02 ^c^	1.20 ± 0.61
% Hydrolyzed	n/a	3	35	8	26	26	14	19 ± 12
**Coumaroyl feruloyl putrescine (CFP)** (mg FAE 100 g^−1^ dw)								
CFP	SF	0.82 ± 0.06 ^ab^	0.48 ± 0.15 ^cd^	0.66 ± 0.02 ^bc^	0.98 ± 0.05 ^b^	0.52 ± 0.08 ^bc^	0.20 ± 0.04 ^d^	0.61 ± 0.27
Non-hydrolyzed CFP	SHF	0.64 ± 0.003 ^a^	0.34 ± 0.03 ^bc^	0.59 ± 0.07 ^ab^	0.73 ± 0.14 ^a^	0.45 ± 0.08 ^ab^	0.15 ± 0.01^c^	0.48 ± 0.21
% Hydrolyzed	n/a	22	29	11	25	13	27	21 ± 7
**Dicoumaroyl spermidine (DCS)** (mg pCAE 100 g^−1^ dw)								
DCS	SF	0.71 ± 0.04 ^a^	0.51 ± 0.10 ^ab^	0.64 ± 0.04 ^ab^	0.45 ± 0.04 ^b^	0.48 ± 0.07 ^ab^	0.15 ± 0.05 ^c^	0.49 ± 0.19
Non-hydrolyzed DCS	SHF	0.10 ± 0.04 ^a^	0.07 ± 0.002 ^a^	0.09 ± 0.004 ^a^	0.09 ± 0.01 ^a^	0.10 ± 0.02 ^a^	0.05 ± 0.0001 ^a^	0.08 ± 0.02
% Hydrolyzed	n/a	86	87	86	80	79	67	81 ± 7
**Dehydrodiferulic acids (DFA)** (mg FAE 100 g^−1^ dw)								
8-O-4′-DFA	IF	8.42 ± 0.08 ^a^	6.88 ± 1.09 ^a^	7.62 ± 0.73 ^a^	6.72 ± 1.14 ^a^	6.09 ± 0.05 ^a^	3.04 ± 0.20 ^b^	6.46 ± 1.86
5-5′-DFA	IF	3.27 ± 0.06 ^a^	2.01 ± 0.84 ^ab^	3.03 ± 0.34 ^a^	2.60 ± 0.45 ^ab^	2.55 ± 0.03 ^ab^	1.29 ± 0.11 ^b^	2.46 ± 0.72
8-5′-DFA	IF	2.90 ± 0.12 ^a^	2.72 ± 0.59 ^a^	2.84 ± 0.34 ^a^	2.60 ± 0.25 ^a^	2.43 ± 0.09 ^a^	1.01 ± 0.11 ^b^	2.42 ± 0.71

**Table 5:** Correlation coefficients among *broas* variables, as described in Table 1. Very strong correlations (|*R*| > 0.8) are highlighted in bold. *p*-Value corresponds to the significance level of Pearson correlation coefficient indicated as *: significant at *p* < 0.05; **: significant at *p* < 0.01.

		SF							SHF-SF		IF						
		PCs	AAs	FAf	pCAf	DFP	CFP	DCS	FAc	pCAc	PCi	AAi	FAi	pCAi	8O4DFA	55DFA	85DFA
SF	**PCs**		0.397	0.492	0.249	0.579	0.566	0.314	0.327	−0.323	0.225	0.291	0.431	−0.103	0.349	0.493	0.405
	**AAs**			0.864 *	0.908 *	0.744	0.795	0.894 *	0.566	0.141	0.830 *	0.847 *	0.993 **	0.832 *	0.959 **	0.794	0.957 **
	**FAf**				0.818 *	0.595	0.719	0.822 *	0.826*	0.426	0.576	0.571	0.915 *	0.545	0.845 *	0.792	0.756
	**pCAf**					0.620	0.678	0.995 **	0.737	0.362	0.736	0.642	0.918 **	0.783	0.986 **	0.910 *	0.940 **
	**DFP**						0.981 **	0.608	0.437	0.025	0.369	0.493	0.735	0.400	0.715	0.750	0.734
	**CFP**							0.663	0.564	0.173	0.377	0.494	0.802	0.426	0.763	0.792	0.746
	**DCS**								0.753	0.330	0.734	0.627	0.908 *	0.742	0.981 **	0.926 **	0.939 **
SHF-SF	**FAc**									0.738	0.183	0.084	0.649	0.231	0.693	0.836 *	0.531
	**pCAc**										−0.217	−0.299	0.215	0.071	0.267	0.367	0.045
IF	**PCi**											0.955 **	0.789	0.897 *	0.770	0.483	0.850 *
	**AAi**												0.797	0.867 *	0.718	0.407	0.813 *
	**FAi**													0.783	0.962 **	0.826 *	0.940 **
	**pCAi**														0.793	0.481	0.820 *
	**8O4DFA**															0.909 *	0.974 **
	**55DFA**																0.851 *
	**85DFA**																

**Table 6:** Amount (%) of phenolic content (PC), antioxidant activity (AA), ferulic acid (FA), *p*-coumaric acid (pCA), hydroxycinnamic acid amides (HCAAs) and dehydrodiferulic acids (DFAs) remaining after raw flour (70% maize + 20% rye + 10% wheat) processing to *broas*.

		Fraction	Samples						Average
			B213	Pig	CV	VA	Fan	Com	
PC	Soluble	SF	153	181	176	176	201	198	181 ± 17 **
	Insoluble	IF	127	115	97	83	83	120	104 ± 19
	Total	SF + IF	133	126	110	99	100	137	118 ± 17 *
AA	Soluble	SF	139	147	138	149	140	128	140 ± 7 **
	Insoluble	IF	123	116	95	99	71	80	97 ± 20
	Total	SF + IF	127	120	103	107	79	88	104 ± 18
FA	Soluble-free	SF	298	350	333	305	353	380	337 ± 31 **
	Soluble-conjugated	SHF-SF	90	57	96	59	76	62	73 ± 17 *
	Total soluble	SHF	109	75	119	75	100	80	93 ± 19
	Insoluble	IF	130	94	91	81	70	123	98 ± 24
	Total	SHF + IF	128	93	92	80	69	119	97 ± 23
pCA	Soluble-free	SF	528	741	525	589	524	395	550 ± 113 **
	Soluble-conjugated	SHF-SF	80	45	72	56	47	95	66 ± 20 *
	Total Soluble	SHF	130	93	127	106	59	143	110 ± 31
	Insoluble	IF	115	110	97	79	60	83	91 ± 21
	Total	SHF + IF	115	108	99	82	59	95	93 ± 20
HCAAs	DFP	SF	96	131	89	107	98	117	106 ± 15
	CFP	SF	84	116	85	90	69	111	92 ± 18
	DCS	SF	100	137	98	91	87	86	100 ± 19
DFAs	8-O-4′-DFA	IF	177	98	100	74	60	110	103 ± 41
	8-5′-DFA	IF	192	71	102	81	58	97	100 ± 48
	5-5′-DFA	IF	173	103	130	102	92	104	117 ± 30

* and **: Significant difference after raw flour processing to *broas* (*p* < 0.05 and *p* < 0.001, respectively).

## Data Availability

The data supporting the findings of this study are available within the article and its supplementary material.

## Supplementary Materials

The following are available online at https://www.mdpi.com/article/10.3390/antiox10050672/s1.
