# Supplementary material for: Broa, an Ethnic Maize Bread, as a Source of Phenolic Compounds
Source: Antioxidants (Basel). 2021 Apr 26;10(5):672. doi: 10.3390/antiox10050672 (PMC8145897; doi:10.3390/antiox10050672)
Supplement: Supplementary file 1 [file antioxidants-10-00672-s001.zip › antioxidants-1180436-supplementary.pdf]

Table S1. Description of the maize flours used in the production of *broas*.

Population	Description
**Broa-213**	Yellow kernel. Early intermediate type. Collect from the farmer in the 2005 expedition to the Central Northern region of Portugal [20].
**Pigarro**	White kernel. Flint type FAO 300 with strong fasciation expression, used in the best soils for human consumption.
**Castro Verde**	Yellow colour. Late flint type FAO 600, with big kernel row number and large ear size.
**Verdeal de Aperrela**	White colour. Late flint type FAO 600, used for breadmaking.
**Fandango**	Yellow colour. Synthetic open-pollinated variety, dent type FAO 600, big kernel row number and large ear size.
**Commercial**	White colour. Nacional Type 175, wholegrain flour.

Table S2. Increase (%) in the antioxidant activity (AA) and phenolic content (PC) after hydrolysis of the soluble compounds (from the soluble [SF] to the soluble-hydrolysed [SHF] fractions) of cereal flours.

*: Significant difference between SF and SHF ($p < 0.001$).

Samples	AA increase (%)	PC increase (%)
Broa-213	89	9
Pigarro	112	15
Castro Verde	72	13
Verdeal de Aperrela	93	27
Fandango	128	11
Commercial	199	21
Average	*115 ± 45 **	*16 ± 7 **
Wheat	*- 25*	*20*
Rye	*12*	*7*

Table S3. Contribution (%) of soluble-free, total soluble and insoluble ferulic (FA) and *p*-coumaric (pCA) acids for the antioxidant activity of the soluble (SF), soluble-hydrolysed (SHF) and insoluble fractions (IF) of cereal flours extracts, respectively.

Sample	Soluble fraction (SF)			Soluble-hydrolysed fraction (SHF)			Insoluble fraction (IF)		
	FA	pCA	Others	FA	pCA	Others	FA	pCA	Others
Broa-213	0.6	0.4	99.0	6.5	2.8	90.7	44.0	6.1	49.9
Pigarro	0.5	0.3	99.2	5.9	2.5	91.6	40.8	6.1	53.0
Castro Verde	0.5	0.5	99.0	5.9	3.1	91.1	47.4	6.4	46.2
Verdeal de Aperrela	0.6	0.4	99.0	7.8	2.6	89.6	50.7	5.5	43.7
Fandango	0.5	0.4	99.0	5.6	2.1	92.3	41.4	4.0	54.6
Commercial	0.6	0.6	98.8	6.2	2.1	91.7	37.1	2.9	60.0
Average	*0.6 ± 0.1*	*0.4 ± 0.1*	*99.0 ± 0.1*	*6.3 ± 0.8*	*2.5 ± 0.4*	*91.2 ± 0.9*	*43.6 ± 4.9*	*5.2 ± 1.4*	*51.3 ± 5.9*
Wheat	0.9	0.1	99.0	4.3	0.2	95.5	15.9	0.6	83.5
Rye	1.0	0.4	98.7	4.5	1.3	94.2	15.0	1.7	83.3

Others: other compounds or synergistic effects; FA: 15.6 μmol TE mg^{-1}; pCA: 23 μmol TE mg^{-1}.

Table S4. Increase (%) in the antioxidant activity (AA) and phenolic content (PC) after hydrolysis of the soluble compounds (from the soluble [SF] to the soluble-hydrolysed [SHF] fractions) of *broas*.

Samples	AA increase (%)	PC increase (%)
Broa-213	24	- 2
Pigarro	39	17
Castro Verde	62	27
Verdeal de Aperrela	37	20
Fandango	16	- 7
Commercial	91	0
Average	45 ± 28 *	9 ± 14

*: Significant difference between SF and SHF ($p < 0.001$).

Table S5. Contribution (%) of soluble-free, total soluble and insoluble ferulic (FA) and *p*-coumaric (pCA) acids for the antioxidant activity of the soluble (SF), soluble-hydrolysed (SHF) and insoluble fractions (IF) of *broas* extracts, respectively.

**Sample**	**Soluble fraction (SF)**			**Soluble-hydrolysed fraction (SHF)**			**Insoluble fraction (IF)**		
	FA	pCA	Others	FA	pCA	Others	FA	pCA	Others
Broa-213	1.4	1.5	97.0	6.7	3.3	90.1	44.6	5.4	50.0
Pigarro	1.4	1.4	97.2	3.9	2.0	94.1	32.4	5.6	62.0
Castro Verde	1.4	1.7	96.9	4.6	2.4	93.0	43.8	6.2	50.0
Verdeal de Aperrela	1.4	1.4	97.2	4.7	2.1	93.2	39.8	4.3	55.9
Fandango	1.6	1.5	96.9	6.6	2.7	90.7	40.3	3.3	56.4
Commercial	2.0	1.7	96.3	4.8	2.6	92.7	54.4	2.9	42.8
Average	*1.5 ± 0.3*	*1.5 ± 0.1*	*96.9 ± 0.3*	*5.2 ± 1.1*	*2.5 ± 0.5*	*92.3 ± 1.6*	*42.5 ± 7.2*	*4.6 ± 1.4*	*52.8 ± 6.7*

Others: other compounds or synergistic effects. FA: 15.6 μmol TE mg^{-1}; pCA: 23 μmol TE mg^{-1}; TE: Trolox equivalents.

Table S6. Calculated values obtained for raw flours (RF = 70% maize + 20% rye + 10% wheat) and used for direct comparison with the corresponding *broas*.

**Variable**		**Fraction**	**Samples (raw flours)**					
			Broa-213	Pigarro	Castro Verde	Verdeal de Aperrela	Fandango	Commercial
PC	**PCs**	SF	37.3	29.6	30.3	32.9	29.7	26.3
	**PCi**	IF	117	147	149	165	174	89.1
AA	**AAs**	SF	0.71	0.62	0.62	0.61	0.56	0.42
	**AAi**	IF	2.53	3.42	2.97	3.22	4.16	2.20
FA	**FAf**	SF	0.31	0.23	0.24	0.27	0.22	0.19
	**FAc**	SHF-SF	4.78	4.30	3.44	4.90	3.89	3.96
	**FAi**	IF	68.6	86.9	86.9	101.3	107.9	50.2
pCA	**pCAf**	SF	0.13	0.08	0.12	0.09	0.10	0.10
	**pCAc**	SHF-SF	1.33	1.18	1.14	1.07	0.91	0.82
	**pCAi**	IF	6.37	8.79	7.93	7.45	7.11	2.64
HCAAs	**DCS**	SF	0.71	0.37	0.65	0.50	0.55	0.18
	**CFP**	SF	0.98	0.41	0.78	1.09	0.75	0.18
	**DFP**	SF	1.83	0.87	1.91	2.36	1.36	0.33
DFAs	**8-O-4′-**	IF	4.85	7.09	7.69	9.10	10.21	2.91
	**8-5′-**	IF	1.72	2.69	2.24	2.61	2.69	1.06
	**5-5′-**	IF	1.73	2.85	3.00	3.24	4.42	1.37

SF: soluble fraction; SHF: soluble-hydrolysed fraction; IF: insoluble fraction; PCs: soluble phenolic content; AAs: soluble antioxidant activity; FAf: soluble-free ferulic acid; pCAf: soluble-free *p*-coumaric acid; FAc: soluble-conjugated ferulic acid; pCAc: soluble-conjugated *p*-coumaric acid; DFP: diferuloyl putrescine; DCS: dicoumaroyl spermidine; CFP: coumaroyl feruloyl putrescine; PCi: insoluble phenolic content; AAi: insoluble antioxidant activity; FAi: insoluble ferulic acid; pCAi: insoluble *p*-coumaric acid; 8O4DFA: 8-O-4′-dehydrodiferulic acid; 85DFA: 8-5′-DFA; 55DFA: 5-5′-DFA.

Values reported as: PC: mg GAE (gallic acid equivalents) 100 g^{-1} dry weight (dw); AA: mmol of TE (Trolox equivalents) 100 g^{-1} dw; pCA and FA: mg 100 g^{-1} dw; DCS: mg pCAE (pCA equivalents) 100 g^{-1} dw; CFP, DFP, and DFAs: mg FAE (FA equivalents) 100 g^{-1} dw.
